# Photothermal therapy using graphene quantum dots

**DOI:** 10.1063/5.0160324

**Published:** 2023-08-21

**Authors:** Mohammad Suhaan Dar, Tanveer A. Tabish, Nanasaheb D. Thorat, G. Swati, Niroj Kumar Sahu

**Affiliations:** 1 Centre for Nanotechnology Research, Vellore Institute of Technology, Vellore 632014, India; 2 School of Bio-Sciences and Technology, Vellore Institute of Technology, Vellore 632014, India; 3 Division of Cardiovascular Medicine, Radcliffe Department of Medicine, University of Oxford, Oxford OX3 7BN, United Kingdom; 4 Nuffield Department of Women's and Reproductive Health, Medical Science Division, John Radcliffe Hospital, University of Oxford, Oxford OX3 9DU, United Kingdom

## Abstract

The rapid development of powerful anti-oncology medicines have been possible because of advances in nanomedicine. Photothermal therapy (PTT) is a type of treatment wherein nanomaterials absorb the laser energy and convert it into localized heat, thereby causing apoptosis and tumor eradication. PTT is more precise, less hazardous, and easy-to-control in comparison to other interventions such as chemotherapy, photodynamic therapy, and radiation therapy. Over the past decade, various nanomaterials for PTT applications have been reviewed; however, a comprehensive study of graphene quantum dots (GQDs) has been scantly reported. GQDs have received huge attention in healthcare technologies owing to their various excellent properties, such as high water solubility, chemical stability, good biocompatibility, and low toxicity. Motivated by the fascinating scientific discoveries and promising contributions of GQDs to the field of biomedicine, we present a comprehensive overview of recent progress in GQDs for PTT. This review summarizes the properties and synthesis strategies of GQDs including top-down and bottom-up approaches followed by their applications in PTT (alone and in combination with other treatment modalities such as chemotherapy, photodynamic therapy, immunotherapy, and radiotherapy). Furthermore, we also focus on the systematic study of *in vitro* and *in vivo* toxicities of GQDs triggered by PTT. Moreover, an overview of PTT along with the synergetic application used with GQDs for tumor eradication are discussed in detail. Finally, directions, possibilities, and limitations are described to encourage more research, which will lead to new treatments and better health care and bring people closer to the peak of human well-being.

## I. INTRODUCTION

Tumors have been one of the most serious risks to human health for decades. As per the study conducted by the International Agency for Research on Cancer, 16–18 × 10^6^ instances of malignancy will be diagnosed each year, with 60% of these occurring in underdeveloped nations.^1^ Surgery, chemotherapies, and radiotherapies are the mainstays of modern tumor treatments. Surgery alone, as it is understood, cannot fully eliminate all tumor cells from the body system. Chemotherapy is often used as a primary treatment for late-stage cancer, or as a substitute for surgery at an early stage cancer, causing significant systemic damage and nonspecific cytotoxicity in both malignant and surrounding non-diseased healthy cells/tissues.^2,3^ Radiation therapy is a treatment for malignant tumors that kills harmful tumor cells with high-energy beams (x-rays) while also harming nearby healthy cells and organs.^4^

Photothermal therapy (PTT) is less toxic than chemotherapy, photodynamic therapy, and radiation therapy. The process of PTT contains nanomaterials as photothermal agents (also termed as photosensitizers), which are directly exposed to the near-infrared (NIR) range, producing local heat and damaging the cancerous cells.^5^ The essential photothermal agents that have been used in this field are gold nanoparticles (AuNPs), nanocages, silver NPs, carbon nanotubes, reduced graphene, graphene quantum dots (GQDs), polydopamine, polyaniline, cetyl palmitate, etc.^6^ These photothermal agents have been extensively used for PTT because of their unique electrical, thermal, size-dependent optical, and magnetic properties.^7^ Carbon-based materials are abundant and play an important part in human development. Since the Nobel Prize was awarded to the discovery of graphene in 2010, it has become a hot topic of research. Scientists have spent the last decade deciphering the biological roles of GQDs and expanding their biomedical applications. GQDs are derivates of graphene and have gained a tremendous attention due to their remarkable biological properties like biocompatibility, minimal toxicity, large surface area to volume ratios, good solubility, surface grafting, and inertness.^8–10^

Photoluminescence is an important property of GQDs, wherein the emission of light energy occurs due excitation of electrons, followed by the subsequent relaxation of excited electrons to a lower energy state.^11^ The excellent photostability and tunable photoluminescence (PL) make them ideal materials for PTT and facilitate *in vivo* distribution by visualizing tumors in mouse models for monitoring therapeutic responses to tumors. Shi and co-workers developed a graphene-based multi-functional nanocomposite with gold and polyethylene glycol (PEG) and found that a photothermal agent acts strongly with negligible cell toxicity and good fluorescence *in vivo*. Therefore, the nanoprobe performed efficiently as an excellent theranostic probe for tumor ablation.^12^ Additionally, Wang *et al.*, prepared GQDs with polydopamine and Prussian blue nanocubes by chemical synthesis. Furthermore, they observed high photothermal conversion efficacy, tremendous bioimaging ability, and efficiently illuminated tumors.

However, large-surface-area nano-sized GQDs could be used as photothermal agents for organ clearance, although there are limitations as well, and the results are more promising than other nanomaterials.^13^ GQDs are more biocompatible and easier to functionalize than traditional bright quantum dots, and they have a lot of potential in PTT and other biomedical applications. The functional groups present on the surface of GQDs are a result of oxidation and passivating features during synthesis, and these polar elements confer excellent aqueous solubility on the GQDs, which is useful for synergetic applications for tumor suppression.^14,15^

The main focus of this review is to cover a basic understanding of GQDs, their properties, synthesis, highlights on representative *in vitro* and *in vivo* experiments, and their application in PTT, specifically to fight against cancer, with other beneficial synergetic cancer therapies, such as chemotherapy therapy, gene therapy, immunotherapy, photodynamic, and radiotherapy.

## II. PHOTOTHERMAL THERAPY (PTT)

**FIG. 1.**
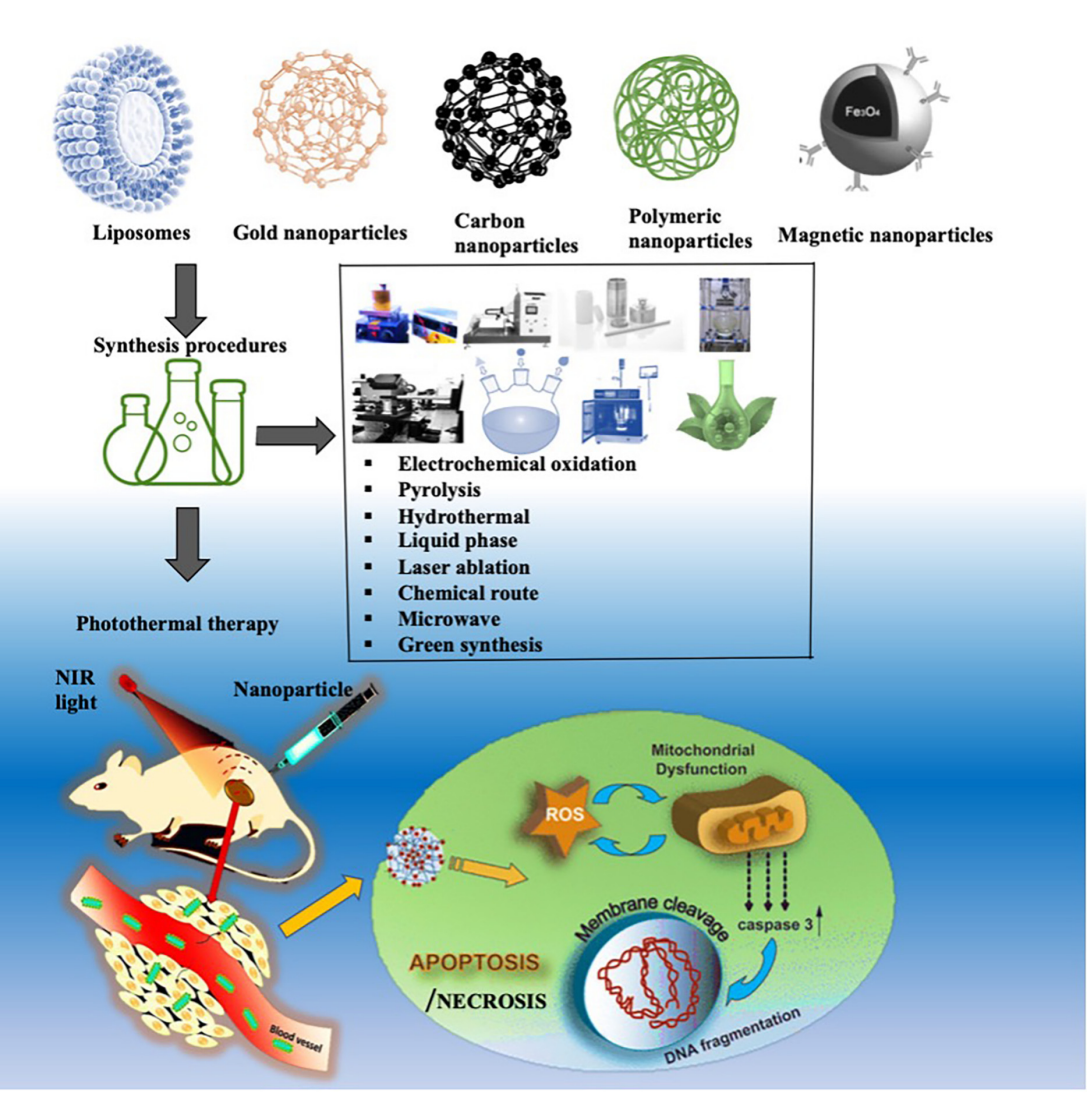
Schematic process of photothermal therapy with nanomaterials and effect on tumor cells when NIR radiation is used.

PTT is a type of therapy that utilizes NPs to absorb and transform laser energy into local heat when exposed to light, resulting in irreversible cell death and tumor elimination. Several nanomaterials, such as gold, silver, carbon, and liposomes, have been used as PTT agents, as shown in Fig. 1. PTT triggered by NIR light occurs in the tumor and is then subjected to NPs' resonance energy, which produces perfectly synchronized vibrations of NPs' conduction-band electrons, producing heat to avoid the severe infection-related consequences, which typically follow surgery, as well as the harmful chemical side effects associated with chemotherapy, with reduced invasiveness and no visible damage to normal tissue.^16,17^ Heating sources, such as NIR or visible light, the magnetic field, ultrasound waves, microwaves, and radio frequency waves, are used to generate a progressive temperature rise in a specified target region, clinically known as hyperthermia.^18^

The optical absorption of most photothermal conversion nanomaterials is currently in the first NIR window, between 700 and 950 nm. Despite great advancements in the NIR-I range, the scattering and absorption of light in specific cells and organs lowers the amount of light of NIR laser on tumor locations dramatically. Therefore, it is desirable to implement NIR light to the NIR-II between 1000 and 1700 nm to maximize the penetration depth of incoming light, create deeper tumor destruction, and improve the therapeutic efficacy of PTT.^19^

### A. Cell death patterns triggered by PTT

The aggregated NPs inside tumors can absorb and convert the energies of photons to heat, which can lead to local and regional hyperthermia. The high temperature can affect the permeability of the cell membrane and its activities, resulting in necrosis or apoptosis of some structural and enzymatic proteins within tumor cells.^20^ Necrosis and apoptosis are possible ways of cell death caused by PTT. In contrast, primary necrosis disrupts the integrity of the plasma membrane to produce damage-associated molecular patterns (DAMPs) and leads to inflammatory responses and immunogenic reactions. In comparison, throughout the process of apoptosis, cell membrane integrity has been maintained and signals such as phosphatidylserine (PS) travel to the extracellular region of the membrane to indicate the phagocytosis of cells. Apoptotic cells are changed when faced with phagocytes in a way that decreases inflammation, a distinct and more appealing response than what happens during necrosis.^21^ Necrosis has been the most prevalent *in vitro* cell response to PTT to date. However, some studies have shown that apoptosis is the main mechanism for cell death at specific levels of light exposure.

High-energy radiation can lead specifically to necrosis, while low-energy radiation can trigger apoptosis. PTT can suppress vascular endothelial growth factors, limit the endothelial proliferation of tumor vasculature, and disrupt the remodeling of the extracellular matrix, leading to a reduction in tumor development and metastasis.^22,23^ In addition, CA4P tubulin disturbances may potentially cause tumors to modify their endothelial morphology, which can rapidly shut down the blood flow around the tumor. Clinical development has been undergoing anti-angiogenesis (CA4P) therapy for the treatment of cervical and other cancers.^24,25^ This will allow researchers to combine the CA4P with photothermal therapy and other therapies for better results in cancer therapy.

### B. Mechanisms of photothermal conversion

The photothermal effect is usually characterized as the rise in temperature caused by light absorption in the material. The ability of different materials to turn light into heat varies depending on how their electrical or bandgap structures respond to electromagnetic radiation.^26^ Thermodynamic vibrations, nonradiative relaxation, and plasmonic localized heating are the three different mechanisms that constitute the photothermal conversion process.^27^ All carbon nanomaterials are suitable for photothermal materials because they are chemically stable, absorb a wide range of light, are light, and are economical. Their ability to convert light into heat depends on the stimulation of loosely bound π electrons and relaxation to those states.^28,29^ However, when considering GQDs, the photothermal thermal conversion efficiency has reached 62.53%.^30^ Moreover, many obstacles will need to be addressed in the near future in order to significantly improve the performance of photothermal materials, such as maintaining the stability of photothermal efficiencies, changing synthesis techniques, and developing cost-effective mass manufacturing methods.^31,32^

## III. GRAPHENE QUANTUM DOTS (GQDS)

### A. Detailed overview of GQDs

**FIG. 2.**
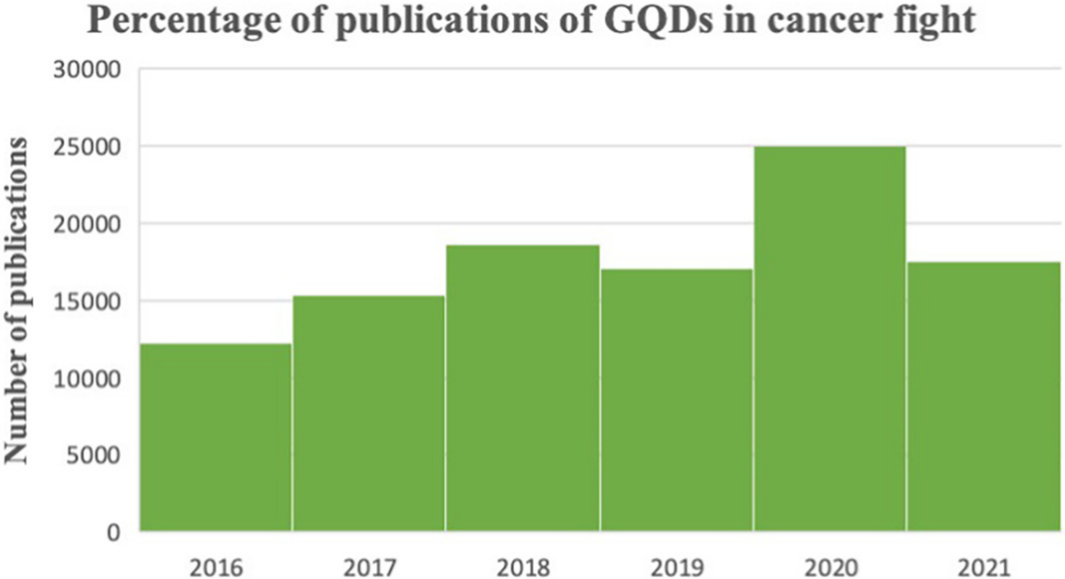
Number of scientific publications per year on GQDs to fight against cancer. Data obtained from Web of Science accessed on March 2023 using the words “GQDs fight against cancer.”

Carbon-based materials are extensively used and play an important role in the modern world. A quantum dot (QD) is defined as a zero-dimensional nanostructure or region that includes quantum confinement in all three spatial directions.^33^ The structural differences between graphene, graphene-oxide, reduced-graphene, GQDs, and fluorographene have significant implications for the properties observed in these carbon-based molecules. The remarkable photochemical stability and size-dependent optical properties of semiconductor QDs, which originate from the quantum-confinement effect, have recently attracted a great deal of attention.^34,35^ Moreover, these materials exhibit high quantum yield, superior photostability, and stable photoluminescence, which distinguish them from conventional nanomaterials, thus making them viable biological materials. In 2008, Ponomarenko and Geim invented GQDs, building on previous work on carbon dots (CDs) from 2004.^36–38^ GQDs are low-toxicity, zero-dimensional fluorescent nanomaterials made up of small graphene fragments ranging from 1 to 100 nm. Graphene is a zero-bandgap material, and GQDs exhibit a tunable bandgap owing to quantum confinement and the edge effect.^39^ GQDs are graphene nanoparticles with the properties of quantum dots. Because of their configurable bandgap, surface imperfections, and zigzag edges, they exhibit a variable fluorescence feature, which lacks graphene.^40^ GQDs employ the property of photoluminescence (PL), which explains the emission of light produced by the excitation of electrons. The number of scientific publications per year on GQDs to fight against cancer have been summarized in Fig. 2.

Although GQDs have attractive features that make them suitable nanomaterials for therapeutic applications, scientists are looking for other alternatives to increase their properties, such as quantum yield and size tenability.^41^ Controlling the size of GQDs, modifying their surface characteristics, or adding dopants like aluminum, nitrogen, or boron can change the PL excitation, emission wavelengths, and enhance different properties.^42,43^ For example, Lee *et al.* utilized benzylamine and 4,4′-(1,2-diphenylethene-1,2-diyl)diphenol (TPE-DOH) with GQDs to prevent aggregation-caused quenching.^44^ Similar to this, Wang H functionalized GQDs with nitrogen doping, increasing quantum yield up to 54% and improving photoluminescence.^45^ Moreover, Ji *et al.* successfully coupled GQDs with PEG to increase colloidal stability and minimize cytotoxicity.^46^ In contrast to dopants, oxygen-rich functional groups confer strong water solubility and facilitate surface conjugation, both of which are useful in biological applications.^47^ Furthermore, the presence of π-orbitals throughout the sp2-hybridized GQDs lattice improves drug delivery.^48,49^ Therefore, GQDs are potentially promising materials for biomedical applications.

## IV. SYNTHESIS STRATEGIES FOR GQDs

**FIG. 3.**
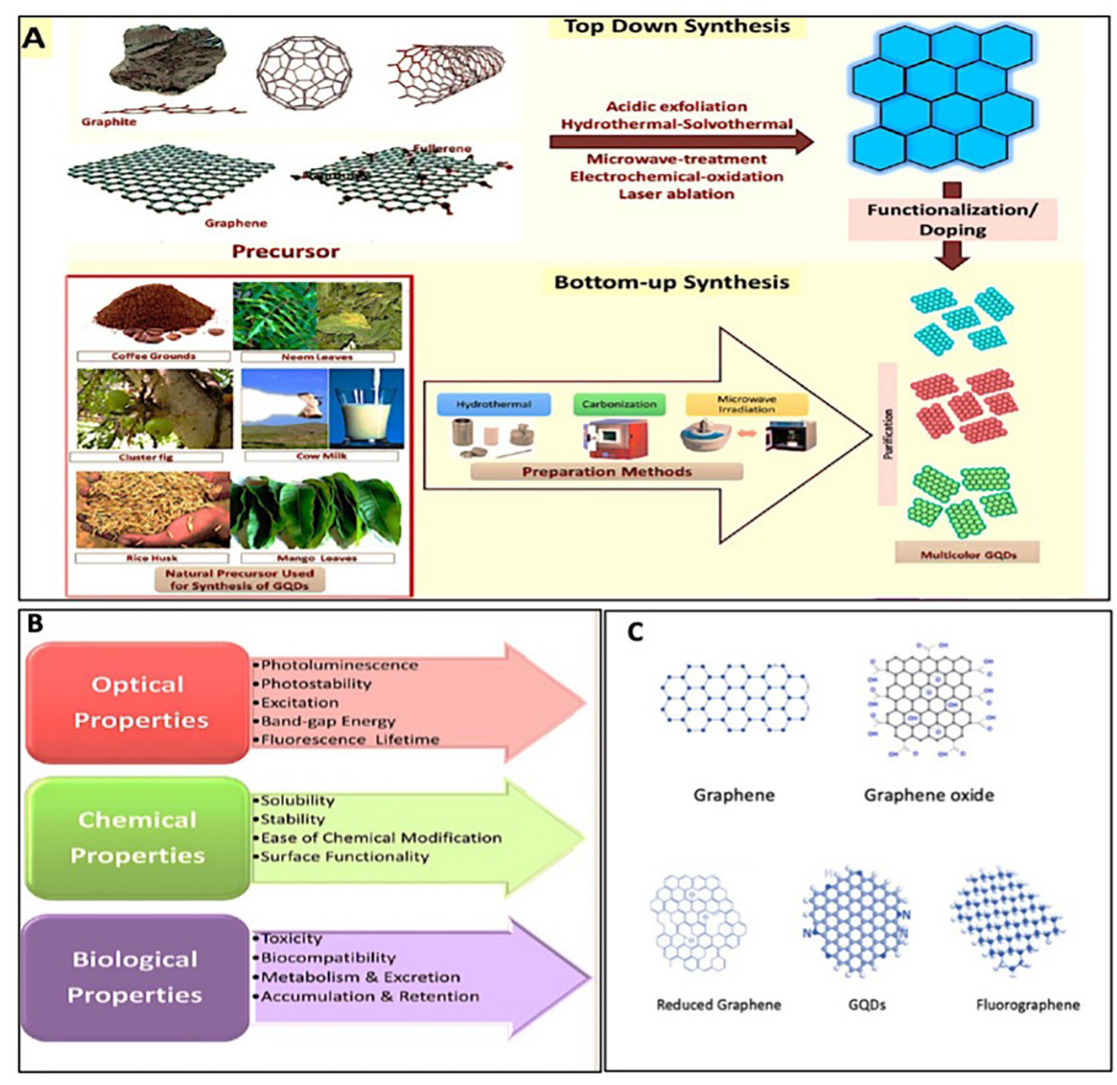
(a) Schematic diagram of the top-down and bottom-up methods for synthesizing GQDs. (b) Different physiochemical properties of GQDs. Reprinted/reproduced with permission from Rahul *et al.,* ACS Biomater. Sci. Eng. **6**(11), 23739878 (2020). Copyright 2020 American Chemical Society.^57^ (c) Structural variations between graphene, graphene oxide, reduced graphene, GQDs, and fluorographene.

GQDs are widely fabricated using a number of synthetic processes, ensuring their solubility and cytotoxicity before being investigated in biomedical applications. GQD's synthesis methods can be classified into two categories: top-down strategy and bottom-up approach. The properties of fabricated GQDs can be altered by utilizing various synthetic methods and tuning reaction conditions. Top-down synthesis is easy and successful and thereby is commonly used in the synthesis process; nanoparticles are obtained by cleaving carbon materials, such as graphene, graphene sheets, GO, fullerenes, and buckytubes, using chemical or physical processes like hydrothermal or solvothermal mechanisms,^50^ electrochemical oxidation,^51^ ultrasonic-assisted or microwave-assisted processes,^52^ oxidative cleavage,^53^ and chemical vapor deposition (CVD), to create GQDs and also acquires dynamic functional groups that help with biological processes.^54^ While in the bottom-up method, GQDs are manufactured using the carbonization method by drying and carbonizing appropriate small molecules or polymers.^55^ The procedure is complex and time-consuming to produce GQDs in large quantities. However, it produces GQDs with fewer surface defects and more precise morphologies and size control.^56^ Figure 3 shows the development of GQDs and the interplay between structural components and their properties by optimizing their functionalization for their use in medicine.

### A. Top-down strategy

#### 1. Hydro/solvothermal synthesis

Hydrothermal/solvothermal synthesis is one of the most popular methods for producing GQDs, in which carbon precursors such as graphite, fullerenes, and graphene sheets are oxidized and then treated at high temperatures to produce GQDs.^58,59^ Graphene oxide (GO) is employed as a precursor material to synthesize GQDs in a number of research by this method.^58,60,61^ Early developments in hydrothermal synthesis have tried to simplify the process while also minimizing environmental damage by avoiding the use of powerful oxidizing chemicals. The procedure, however, does not allow for exact control of size, morphology, or other features.^62^ Recent attempts to overcome these issues have already been made, with the goal of creating GQDs in a simple and controlled manner in one step by modifying the control parameters.

#### 2. Oxidative cleavage

Oxidative cleavage involves oxidation cutting of carbon bonds, and it is the most extensively used approach. H_2_SO_4_, HNO_3_, or other oxidizers are used in this procedure to break carbon-carbon bonds like graphene, GO, and carbon nanotubes.^63^ In a study by Shin *et al.*, they conducted the first step for 60 min, mixing GO with an organic specific solvent and oxone. They used sonication or UV-irradiation to make GQDs.^64^ Furthermore, additional studies can shed light on how to get around things like burning, complexity, and exposure to effectively determine the limitations and strengths of green chemistry.

#### 3. Electrochemical oxidation

Electrochemical synthesis offers more precise formation of GQDs than conventional synthesis techniques. Many studies have been performed to develop GQDs using the electrochemical oxidation approach.^65–67^ An electrochemical oxidation method involves the exfoliation of multilayer bulk materials such as carbon nanotubes, graphite rods, and graphite sheets with a uniform shape, size, and high yield using an applied electric field.^38,68^ There are two methods for fabricating material by electrochemical oxidation. One is that electrochemical oxidation immediately breaks the carbon bonds of carbon-containing precursors.^69^ Another way to make GQDs is to oxidize H20 to make hydroxyl or oxygen-free radicals, which can then be oxidized into GQDs.^70^ Furthermore, it is anticipated that the functionality of electrochemically exfoliated GQDs can be improved by changing the electrolyte solution and that other weak electrolytes can be used with carbon-based materials, such as CNTs, carbon fiber, and graphite rods, to boost nanomedicine, nanotechnology, and biomedical fields.

### B. Bottom-up strategy

#### 1. Carbonization/pyrolysis

Carbonization/pyrolysis is an easy and eco-friendly procedure that involves the degradation of organic compounds such as ethanolamine and citric acid, acetylacetone, and amino acids under high temperatures, thus changing their chemical content and structure. The preparation of various forms of GQDs by pyrolysis methods, such as single-doped GQDs functionalization, and multi-doped GQDs is highly dependent on the chosen precursor.^71–73^ However, long purification steps, such as passive dialysis, are frequently included in these systems.^74^ Therefore, pyrolysis will become a more relevant synthesis process for GQDs as reaction conditions improve and effective purification procedures are developed. Moreover, addressing product stream complexity will be beneficial.^75^ In addition, direct carbonization of organic solvents is an important strategy for producing GQDs without using catalysts, or common organic solvents such as toluene, glycerol, methanol, dimethyl formamide, ethyleneglycol, acetone, and ethylenediamine.^76^ Several studies have reported the use of different organic precursors, including ethylene diamine tetraacetic acid (EDTA), glucose, tartaric acid, sucrose, lysine, arginine, and cysteine, demonstrating that the reaction temperatures highly relies on the precursor used.^77–79^

#### 2. Stepwise organic synthesis

Stepwise organic synthesis method employs benzene compounds for fabrication and incorporates GQDs from small carbon precursors via organic reaction, resulting in a precise number of carbon atoms and a consistent shape and size.^80–82^ However, the stepwise organic procedure demands precise environmental conditions and specific substances for the reaction to take place. The process has lost some of its significance in biomedical applications due to the availability of a superior approach.

#### 3. Green synthesis of GQDs

**FIG. 4.**
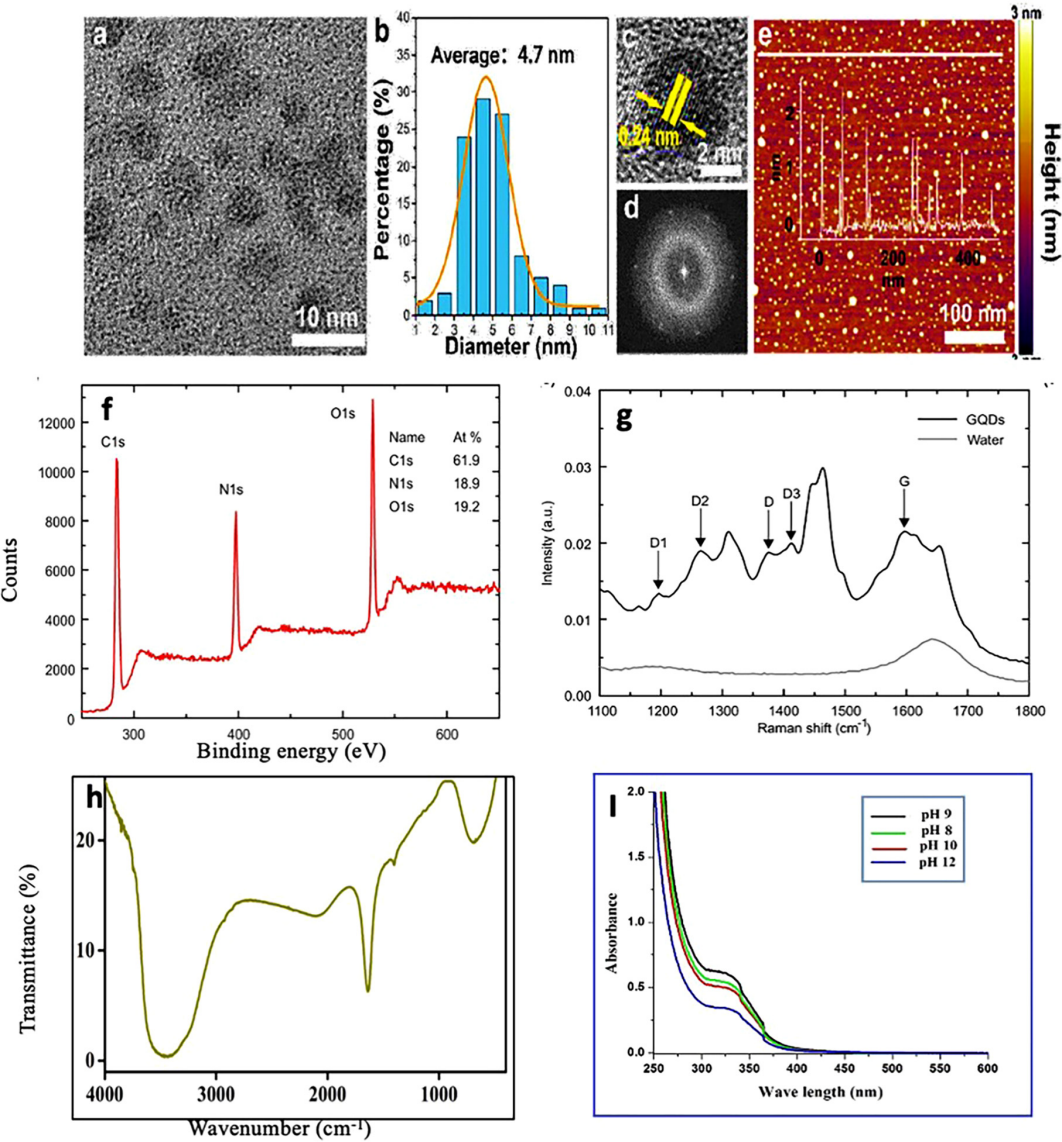
Characterization of GQDs: (a) transmission electron microscopy (TEM) image, (b) particle size distribution, (c) D-spacing of GQDs, (d) fast Fourier Transform (FFT) pattern of GQDs, and (e) AFM image of GQDs. Reprinted with permission from Huang *et al.,* Langmuir **9**(34), 250258 (2017). Copyright 2017 American Chemical Society. (f) X-ray photoelectron spectroscopy (XPS). (g) Raman spectroscopy. Reprinted with permission from Fasbender *et al.,* Sci. Rep. **9**, 2045–2322 (2019). Copyright 2019 Authors, licensed under a Creative Commons Attribution 4.0 License.^87^ (h) Fourier-transform infrared spectroscopy (FTIR). Reprinted with permission from Deng *et al.*, Mater. Res. Express **7**(2), 025517 (2020). Copyright 2020 Author(s).^88^ (i) UV-VIS spectroscopy. Reprinted with permission from Naik *et al.,* J. Nanostruct. Chem. **7**, 8589 (2017). Copyright 2017 Author(s), licensed under a Creative Commons Attribution 4.0 License.

GQDs eco-friendly and low-cost synthesis has recently been the subject of many studies.^83,84^ An emerging domain in nanotechnology is the use of green synthetic pathways, which has astonishingly high QYs (more than 80%), with photoluminescence from blue to red regions, and contributes positively to both the environment and the economy. To produce biomass-derived GQDs, a number of biomaterials have already been proposed, including wheat straw, glucose, wood charcoal, citric acid, rice husk, charcoal, etc.^85,86^ The green methods can offer special insight into the interactions between cells and GQDs, which is extremely helpful for bioimaging and other related biomedical applications, such as drug delivery, photodynamic therapy (PDT), and PTT. Table I highlights the summary of synthesis methods used for the synthesis of GQDs along with their precursor materials, emission wavelength, size, and typical applications. Figure 4 illustrates the basic features of GQD such as shape, size, composition, functional groups, and photoluminescence using characterization tools.

**TABLE I.** Recent synthesis method of GQDs used with PTT. KMnO_4_ —potassium permanganate, NaNO_3_ —sodium nitrate, TEOS —tetraethyl orthosilicate, DMSO —dimethyl sulfoxide, H_2_O_2_ − hydrogen peroxide, H_3_PO_4_ —phosphoric acid, H_2_SO_4_ —sulfuric acid, NaOH —sodium hydroxide, PEI − polyethyleneimine, DMF − dimethylformamide, Zn(NO_3_)_2_ —zinc nitrate, APBA – aminophenylboronic acid, HNO_3_ —nitric acid, NHS — N-hydroxysuccinimide, CTAT —cetyltrimethyl-ammonium tosylate, KOH —potassium hydroxide, NH_3_ —ammonia, Bi (NO_3_) 3.5H_2_O —bismuth nitrate, EDC − 1-Ethyl-3-[3-dimethylaminopropyl]carbodiimide, TEA —triethanolamine, CH_3_OH —methanol, MEH-PPV —poly[2-methoxy-5–2-ethylhexyloxy-1,4-phenylenevinylene, and MWCNTs —multi-walled-carbon nanotubes.

Synthesis method	Material used	Emission peak	Size	Application	Reference
Combinational method, Hummer's method, nano-precipitation, electrostatic interaction	MWCNTs, HNO_3_, Graphite rods, MEH-PPV, PEI, CH_3_OH	500–826 nm	5.2 nm	Effective cancer catalytic agent	89
Sol−gel process with modifications	APTES, TEOS, TEA NHS, CTAT, EDC	481 nm	50 ± 60 nm	Higher efficiency to kill cancer cells	90
Pyrolysis method	NAOH, NH_3_ H_2_O Bi (NO_3_) 3.5H_2_O, KOH, citric acid, HNO_3_	⋯	16.5 nm	CT imaging and cancer theranostics probe	91
Hydrothermal method	APBA, acetone, H_2_O_2_	240–600 nm	5 nm	Imaging-guided cancer therapy and treatment monitoring.	42
Chemical method/general synthetic route	2-Methylimidazole, CH_3_OH, Zn (NO_3_)_2_	⋯	50 ± 100 nm	Anti-cancer drug for synergistic chemo- photothermal therapy	92
Solvothermal method	H_2_O_2_, acetone, DMSO, DMF.	380 nm	3.6 nm	Imaging-guided cancer therapy	93
Solvothermal method	Graphite powder, NaNO_3_, H_2_SO_4_, KMnO_4_	500–600 nm	10 nm	Hyperthermal therapy for cancer treatment	94
Hydrothermal method	Graphite powder, PEI, KMnO_4_, H_3_PO_4_, H_2_SO_4_, NaOH	250–380 nm	5–8 nm	Nanohybrid for cancer treatment	95

## V. PROPERTIES OF GQDS

### A. Optical properties

**FIG. 5.**
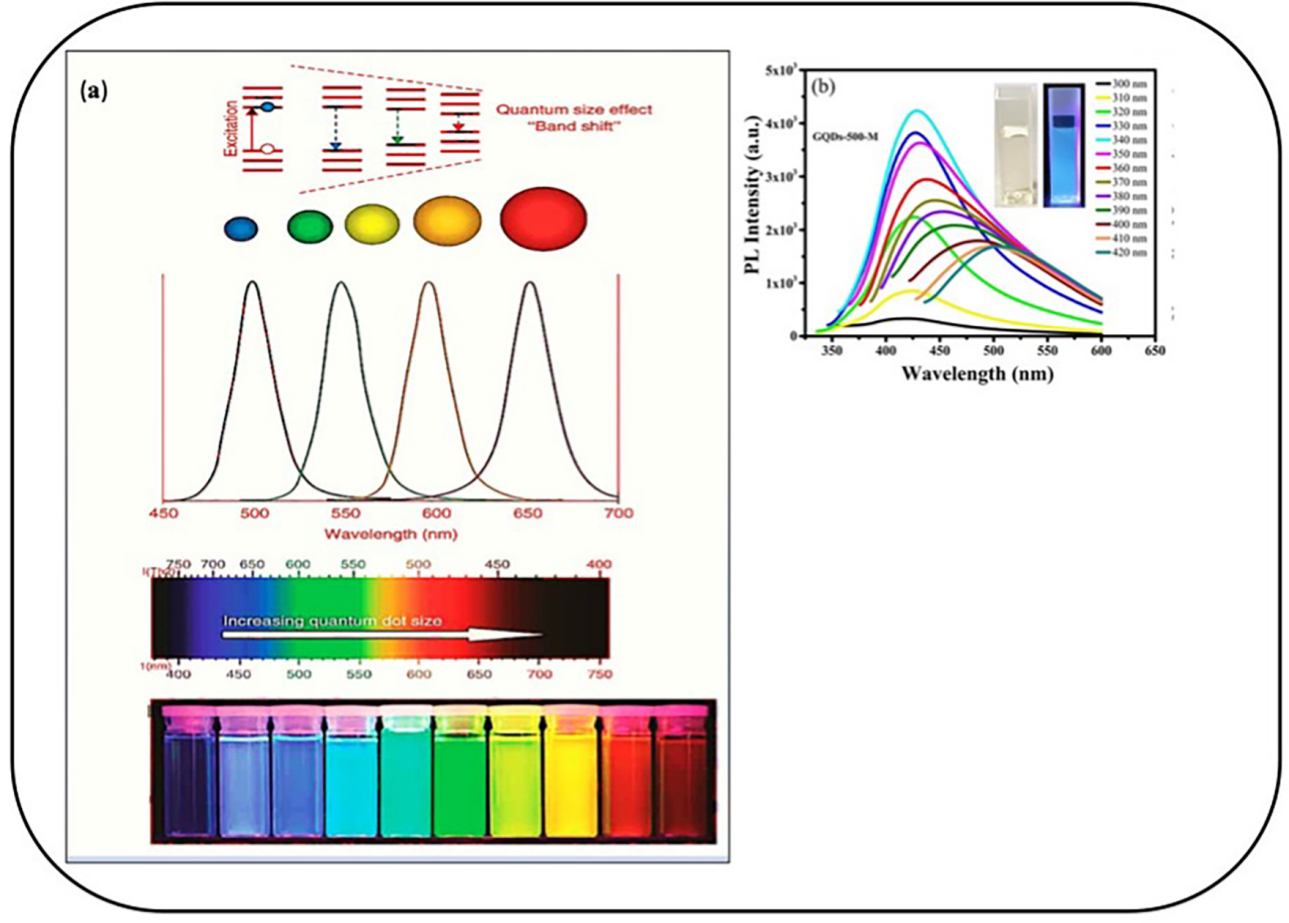
(a) Relationship between optical properties and the size (quantum confinement effect) of GQDs. Reprinted with permission from Mansur, WIREs Nanomed. Nanobiotechnol. **2**, 1939–0041 (2010). Copyright 2010 John Wiley and Sons Clearance Center, Inc.^101^ (b) Photoluminescence spectroscopy of GQDs excited by the light of different wavelengths. Reprinted with permission from Abbas *et al.*, Sci. Rep. **10**, 20452322 (2020). Copyright 2020 Authors, licensed under a Creative Commons Attribution 4.0 License.^102^

GQDs constitute superior crystallinity, which differentiates them from other carbon-based elements. According to reports, the inter-layer space structure ranges from 0.343 to 0.481 nm, which can be modified by adding dopants like nitrogen, aluminum, and boron and functional groups present on the edges, contributing to many applications.^96–99^ However, the structure can be shifted by the extensive sp2 hybridized carbon by the process of quantum confinement, which plays a significant role in photoluminescence (PL), including with the surface edge state.^100^ This has demonstrated excellent results in biomedical applications. Figure 5 shows the relationship between optical properties and the size of GQDs along with the PL spectra, having emission peak at 450 nm being extended from 300 to 600 nm, resulting in a change in luminescence due to the variation in size.

### B. Electronics properties

GQD electronic structure and energy bandgap have been extensively studied with armchair (AM) and zigzag (ZZ) edge group conformations. In graphene, each carbon atom has three bonding orbitals that are sp2-hybridized and have a trigonal planar orbital geometry. The electronic and optical properties of GQDs, such as bandgap and fluorescence, are size and shape-dependent. The PL of GQDs reveals the importance of the bandgap of GQDs.^7,103–105^ As a result, the modification of the intrinsic properties of GQDs is dependent on the accuracy with which their sizes and shapes are controlled. Edge passivation with various functional groups can also change electronic properties.^106^ The conditions of fabrication methods are crucial to accomplishing this task. In this direction, the size influences the bandgap. Biswal and his co-workers reported that GQDs have an average size of 3.5 nm. The GQD bandgap energy was determined to be 2.52 eV using the Tauc plot technique.^107^ Similarly, Ji *et al.* constructed GQDs smaller than 2 nm in size and had a bandgap ranging from 0.1 to 0.6 eV.^108^ Energy gap regulation also benefits from chemical functionalization. Functionalizing hexagonal-armchair GQD edges with O-atoms reduces the enormous energy gap. Alternatively, attaching four nitro groups to the edges reduces the energy gap to 3.01 eV.^109^ Cui *et al.* also reported chemical functionalization alters electron distribution in the conjugated π system and the epoxy groups, impacting bandgaps and excited state properties.^110^ Moreover, quantitative studies have been published to disclose the electrical structure and energy bandgap of GQDs with highly specified dimensions and chemistry.^111–116^

### C. Biocompatibility of GQDs and NIR ranges of PTT

GQDs have high biocompatibility due to their presence of carbon atoms, ultra-small size, and high oxygen content. Biocompatibility refers to a material's capacity to fulfill its essential role without producing unwanted cellular responses.^117^ Comparing GQDs with other 2D biocompatible materials like MXene quantum dots (MQDs), graphitic carbon nitride quantum dots (gCNQDs), and molybdenum disulfide quantum dots (MoS2 QDs), these 2D materials exhibit low cytotoxicity, good stability, greater cell permeability, less toxicity, excellent photostability, and are optically promising. Although extensive research are still required to improve their biodegradability, long-term toxicity, histopathology, and biodistribution.^118–123^ GQDs have great water solubility and give a nontoxic substitute to metal nanoparticles by removing toxicity issues. Some of the obstacles like hydrophobicity, lateral size, oxygen concentration, hydrophilicity, functional groups, and dosage quantity may all influence GQDs cytotoxicity and accumulation in organs, as well as other biological aspects.^124^ The study of GQDs at pharmacokinetic and cellular levels is still in its development. Nevertheless, emerging research has extensively demonstrated the effects of GQDs *in vitro* and *in vivo*, which will be discussed in detail in later sections.

**FIG. 6.**
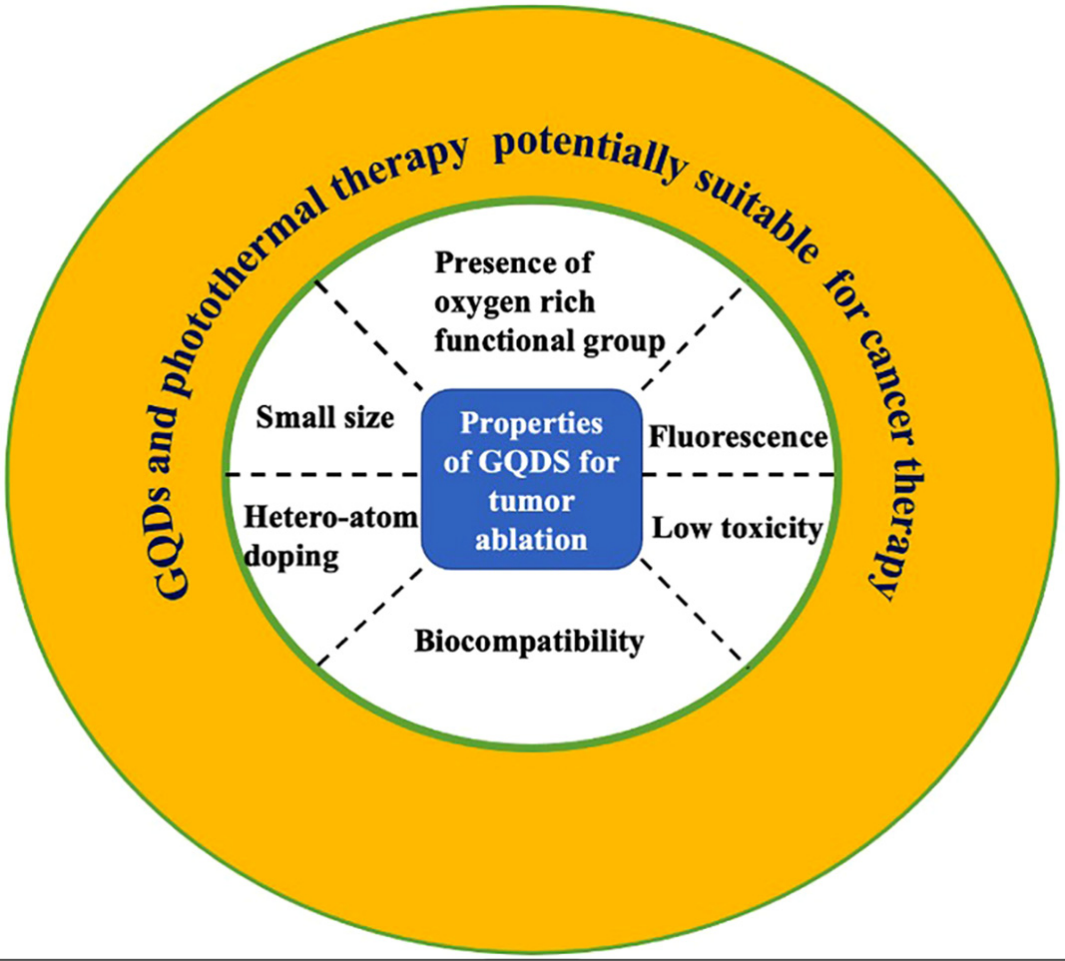
Properties of GQDs suitable with photothermal therapy for tumor suppression.

Since much of the application of GQDs in biomedical studies makes use of the PL property to visualize the interior structure and appropriately detect the tumor spot to eradicate malignancy, it still demands irradiation from a laser source. Figure 6 displays the characteristics of GQDs that are compatible with PTT for tumor suppression. PTT uses various NIR ranges (between 700 and 1700), NIR-I could reduce detection sensitivity, light scattering, and increase the tissue absorption rate since the NIR-I range has some disadvantages like limited energy uptake by water, oxyhemoglobin, and deoxyhemoglobin, which leads to unsatisfactory penetration depth and autofluorescence by endogenous chromophores in the body.^125–128^ Numerous studies have been researched in the NIR-II window to investigate potential therapeutic biomedical applications and NIR-II contrast agents. These include niobium carbide, gold nanostructures, and rare-earth metal nanoparticles (NPs), as well as semiconductor quantum dots.^129–132^

Therefore, it is crucial to create effective photothermal nanomaterials with high NIR-II absorbance. For example, Xu and colleagues demonstrated an outstanding photothermal effect using a high NIR-II-1064 nm laser wavelength with 34% photothermal conversion efficiency, allowing total tumor ablation as well as immunogenic cell death during immune response activation.^133^ Likewise, Wu *et al.* used a 1275 nm laser; their results demonstrate that it is more effective than an 808 nm laser at increasing temperatures *in vitro* and destroying tumors *in vivo*.^134^ Furthermore, Zhang and his team created 1300 nm absorption semiconducting polymer nanoparticles with a photothermal conversion efficiency of 60%, making them an appealing preference for future clinical transformation.^135^ However, these NPs may pose health risks when employed in *in vivo* conditions due to nondegradable nature, poor water solubility, and potential heavy metal toxicity. Recently, GQDs with surface modification have proven to be one of the promising nanoprobes with high chemical stability, photostability, compatibility with hydrophilicity, and no additional surface modification needs in the second window NIR of PTT, with a high photothermal conversion efficiency of nearly 78% at 808 and 1064 nm, respectively.^136–138^

To learn more about the observed phenomenon and how it affects the use of GQD in the real world, we need to do a thorough research and bring different fields to the same shelter.

## VI. JOURNEY OF GQDS INSIDE THE CELLS

**FIG. 7.**
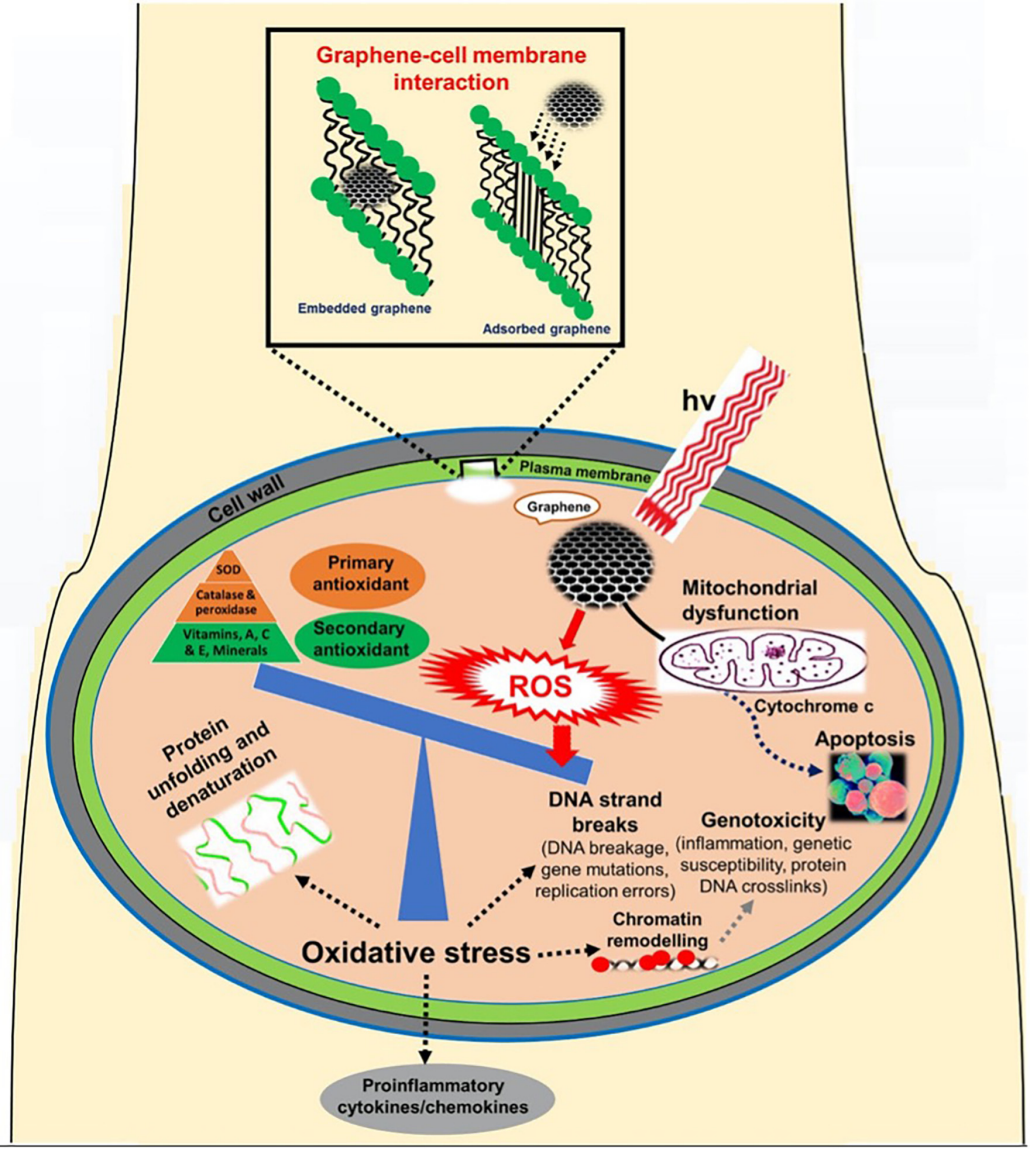
Potential pathways through which reactive oxygen species (ROS) are linked to the cellular toxicity of graphene. Reprinted with permission from Tabish *et al.,* Redox Biol. **15**, 2213–2317 (2018). Copyright 2018 Authors, licensed under a Creative Commons Attribution 4.0 License.

Endocytosis allows nanoparticles to connect with the cell's outer membrane. It is classified into several types based on the cell type, proteins, lipids, and other components involved in the process. GQDs, which are considerably smaller particles, could reach cells through a variety of methods like phagocytosis, clathrin-mediated, and caveolae-mediated in brief; cell uptake is mostly accomplished through endocytosis.^139–141^ A study conducted by Kumawat and co-workers studied the cell cycle differences in L929 cells during various time frames, and they noticed a sudden increase in S-phase at varying intervals of time after 4–8 h, which suggests increased DNA content in the cells than control cells. Within 8 h, the self-assembled sGQDs guided themselves from the cytoplasm to the nucleus and positioned themselves within the nuclear area. The sGQDs entered the cells quickly (within 2 h) and traveled to the nucleus, where they entirely self-localized after 8 h.^142^ In comparison to random large-sized GO nanosheets, similarly, Zhang and colleagues found that ultrasmall GO nanosheets with lateral sizes of less than 50 nm had decreased cytotoxicity and greater cellular absorption. Nevertheless, GQDs, on the other hand, are considerably smaller pieces of GO that could reach cells by a variety of methods.^143^ Another study by Mendes and colleagues suggests that small flakes are ingested via clathrin-mediated endocytosis (CME), while large flakes are internalized via phagocytosis and CME.^144^ Moreover, certain cell uptake inhibitors are employed to investigate each of these methods for GQDs entry and determine how they achieve the journey through these pathways. GQDs also show good mitochondrial targeting abilities; mitochondria are the main source of ROS in living cells and are an important factor of oxidative metabolism and cell death, making them a strong target for PDT as shown in Fig. 7. Additionally, by combining PTT and PDT in one system, the ROS produced in the photodynamic process can inhibit the expression of genes involved in heat generation, thereby boosting the susceptibility of the cancer cells to PTT.^145,146^ A specific type of therapeutic system that targets the mitochondria was proposed in a recent study by Wang and colleagues, and they proved that the nanoprobe may effectively accumulate in the mitochondria of cancer cells, cause apoptotic hyperthermia, and produce enough ROS when exposed to laser light.^147^

### A. *In vitro* and *in vivo* experiments of GQDs with PTT

**FIG. 8.**
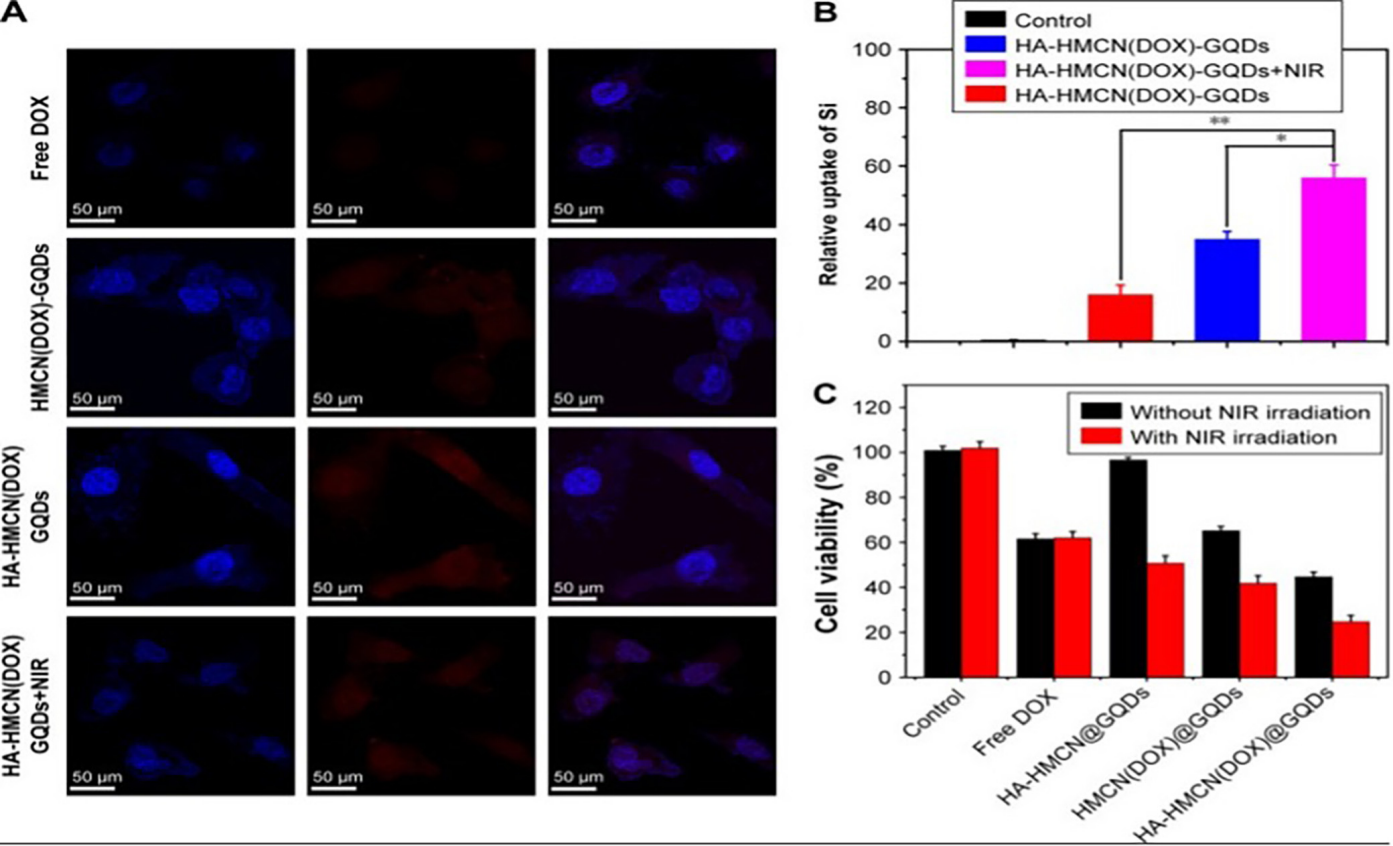
(a) Confocal laser scanning microscopy images of Hela cells treated with various formulations after 4 h incubation (DOX concentration: 5 g/ml). 50 m scale bar. (b) The mass of silicon internalized in HeLa cells after treatment with different formulations. (c) Cell viability of the HeLa cells after 8 h of incubation with different formulation suspensions, with and without NIR irradiation for 5 min. Reprinted with permission from Fang *et al.*, Int. J. Nanomed. **13**, 59916007 (2018). Copyright 2018 Authors, licensed under a Creative Commons Attribution 3.0 License.

**FIG. 9.**
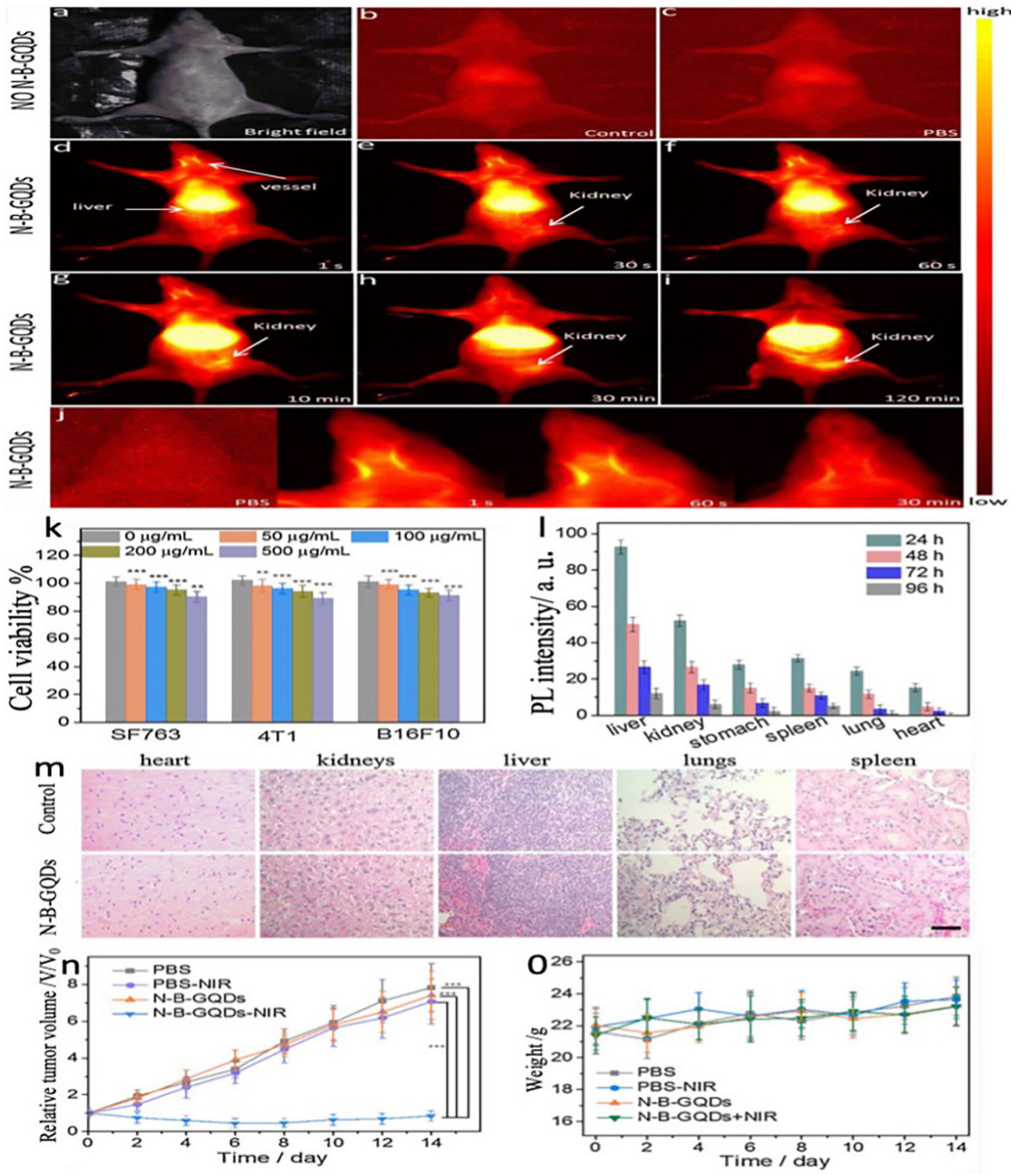
*In vivo* experiment with nude mice administrated an injection of N-B-GQDs with PTT. (a)–(c) NIR-II imaging depicts a nude mouse that did not get an injection and a naked mouse that received PBS saline intravenously. (d)–(i) Nude mouse NIR-II fluorescence illustrations following intravenous N-B-GQDs (1 mg/ml, 200 *μ*l) injection at different intervals. (j) Higher-magnification NIR-II fluorescence images of a nude mouse revealing head blood vessels at various time intervals. (k) N-B-GQD cytotoxicity investigation (*in vitro*). (l) N-B-GQD biodistribution in different tissues and organs of bare mice. (m) Stained nude mice tissue slices with control and N-B-GQDs treatment. (n) Curves of tumor growth and (o) bodyweight shifts in mice treated under different conditions over 14 days. Reprinted with permission from Wang *et al.,* Appl. Mater. Today, **14**, 23529407 (2018). Copyright 2018 Elsevier Clearance Center, Inc.

In recent research, GQDs have shown great biocompatibility and minimal cytotoxicity. GQDs entering the cells can affect apoptosis, cellular division, and many more.^148^ Figure 8 illustrates the *in vitro* images of different formulations of GQDs in Hela cells demonstrating excellent cell viability and tumor elimination using PTT mechanism. However, the mechanism at cellular and sub-cellular levels is not well understood. Some studies have shown that derivates of graphene-like RGO, GO, and doped graphene has shown toxic effects.^149,150^ In contrast, GQDs showed good cell toxicity and viability with good photothermal efficacy.^151^ Wang *et al.* used three different kinds of cancer cell lines to check the cytotoxicity of the nitrogen-boron GQDs, where more than 94% of the cells survived 72 h after treatment of the nanomaterial, while *in vivo* distribution outcomes indicated that a sufficient amount of N-B-GQDs accumulated in the organs but majority were cleared after 96 h post-injection as depicted in Fig. 9. They also pointed out that photothermal therapy for 5 min adequately suppresses tumor growth, thus relieving the concerns of heavy metal toxicity.^42^

Li *et al.*, have confirmed that the cytotoxicity of HeLa cells reached significant levels under 808 nm irradiation. Folic acid was linked with GQDs by a reaction between the amine groups of FA and the carboxyl groups near the edge of GQDs, resulting in FA-functionalized GQDs. They have observed that IR780's water solubility is improved by almost 2400 times, and its encapsulation efficiency is up to 99.36%. Furthermore, IR780 folic acid GQDs are good for producing heat, with 87.9% photothermal conversion efficiency, and IR780 builds up in the organs *in vivo*, but the fluorescence intensity of GQDs was satisfactory.^152^

Hu *et al.*, found that without NIR laser irradiation, the GQDs-semiconducting polymer nanozyme had negligible cytotoxicity, but when treated with NIR laser, the GQD-SPNs exhibited significantly higher cytotoxicity to cancer cells than SPNs, and in *in vivo*, they found the aggregation of GQD-SPNs and SPNs in tumor regions due to the substantial permeability and retention (EPR) effect.^89^ In another study conducted by Prasad *et al*,^153^ under NIR irradiation, GQDs nanostructures with a uniform size of 120 nm may successfully generate heat for improved synergistic photothermal efficiency. Irradiation with a NIR laser may also improve cellular uptake. Within the xenograft mice models, the pharmacokinetics of nanoparticles were measured using DOX fluorescence in blood samples at different intervals. With prolonged blood half-lives, GQDs significantly improved blood circulation. Furthermore, there was no evidence of toxicity or adverse effects *in vitro* or *in vivo*.^153^ However, surface modification and functionalization are significant approaches to improve the characteristics of GQDs in order to boost their potential uses in biomedicine. Meanwhile, the impact of GQDs surface modification on toxicity and function is still lacking due to the variations and complexity of GQDs. As a result, intense research is required to elucidate the interaction of functionalized GQDs with biomolecules such as proteins, DNA, and lipid molecules.

## VII. COMBINATIONAL THERAPIES WITH GQDS AND PTT

**FIG. 10.**
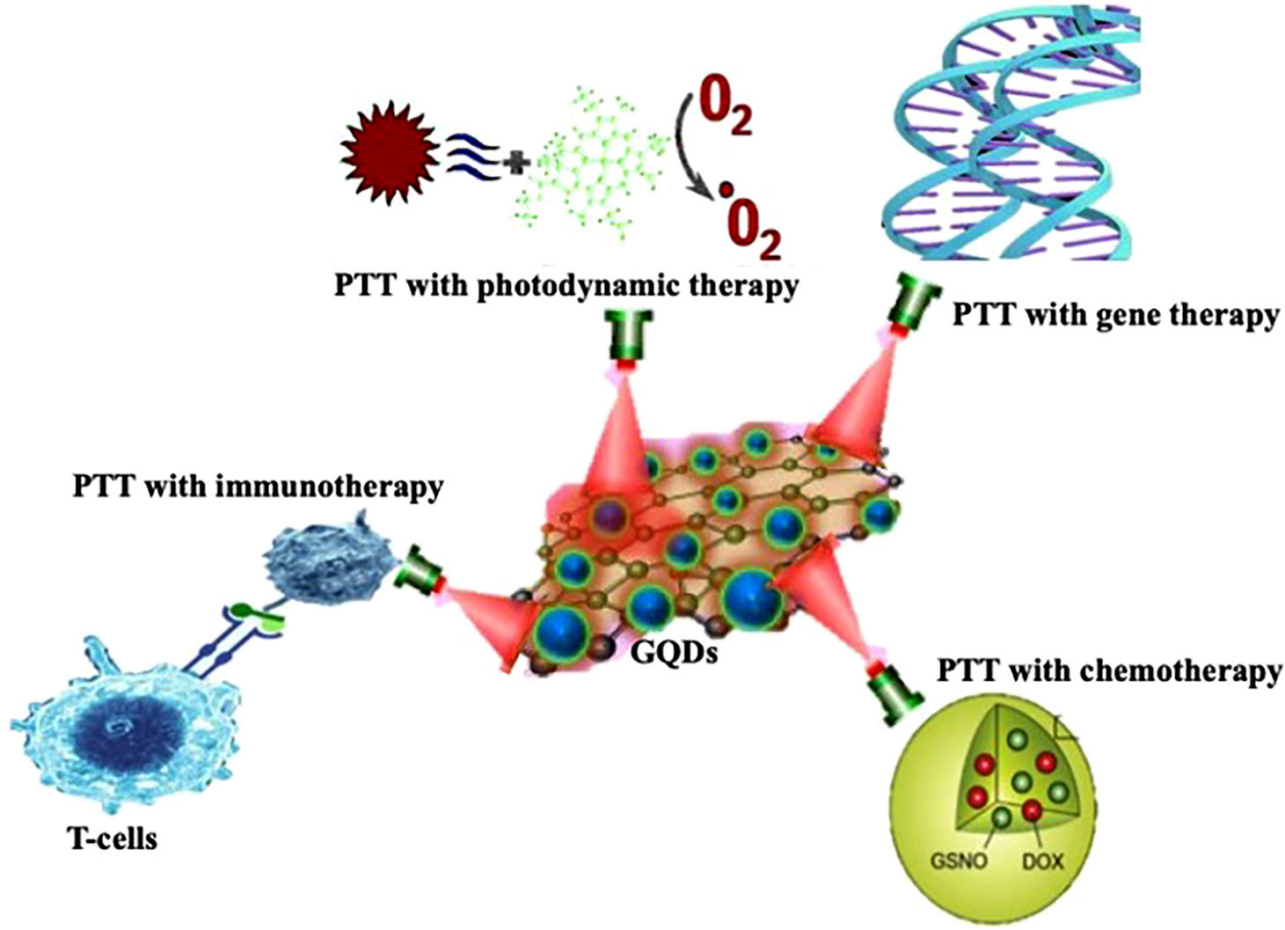
Combinational therapies of GQDs with PTT.

Nanomaterials have been produced and employed in a variety of biomedical applications, including imaging, drug administration, gene therapy, sensing, and photothermal therapy.^154^ Even though nanomaterials have favorable features that make them ideal candidates for biological applications, toxicity, size and distribution, modification, low penetration, and renal clearance are all limitations of nanoparticles, and scientists are actively researching ways to improve their properties.^155,156^ Surface functionalization, polymer capping, core-shell structure, targeting, and biosafety are just a few of the recent breakthroughs that can be used to address these concerns. Moreover, combining PTT with other treatment methods offers an opportunity to leverage the advantages and compensate for the drawbacks of each therapeutic procedure, which led to therapeutic effects that may be additive or synergistic. Figure 10 demonstrates several GQD-PTT combination therapies. In addition, the cooperation between different therapies can lead to anti-tumor impact at low doses of photothermal agents or low laser powers to reduce the potential of nonmalignant tissue toxicity.^157^

### A. GQDs with PTT and gene therapy

The method of curing diseases by transferring nucleic acids to cells is known as gene therapy. Instead of chemotherapy, which inhibits signaling functions and causes tumor tissue death, genetic therapy provides a treatment strategy by using DNA or RNA that can replace a mutant gene, or express new product genes to combat disease.^158^ The key element for the combination of gene therapy and PTT is the light-photothermal effect. Photothermal effects combined with different nuclear therapeutic acids lead to synergistic therapeutic effects while trying to eliminate the target cell and modulating the disease-related gene expression. A gentle photothermal effect makes improved gene therapy easier by overcoming challenges like gene delivery intra-cells, cell phase uptake, endosomal escape, and cytosolic release.^159^ Kim *et al.* found that carbon dots (CD) enhance cellular uptake of siRNA with negligible cytotoxicity and provide fluorescence, thereby inhibiting tumor growth in breast cells.^160^ Similarly, Luo *et al.* reported iron-doped CD with good water solubility and biocompatibility along with photothermal efficacy of 63%, which enhanced the gene transfection both *in vitro* and *in vivo*. Thus, the nanohybrid paved the way for synergetic cancer therapy.^161^ Furthermore, Deng et.al employed four different types of GQDs, of which A-GQDs caused the development block of zebrafish embryos at exposure levels above 100 g/ml, whereas the other three GQDs did not produce any developmental harm even at the highest concentrations.^162^ Although it is quite encouraging as next-generation therapeutics, it has certain limitations, like biocompatibility of gene delivery agents, long-term toxicity, and the bioavailability of photothermal agents^163^

### B. GQDs with PTT and chemotherapy

Chemotherapy is often used as a primary treatment for late-stage cancer or as a substitute for surgery in early stage cancer, causing significant systemic damage and nonspecific cytotoxicity in both malignant and normal cells.^157^ Effective cancer treatments that may eliminate large solid tumors, diffused malignant nodules, and tumor recurrence is highly in demand. PTT is a potential treatment for local tumors that involves the thermal ablation of malignant cells. However, there has been tumor mass found near the treatment margins, and it is hard to get rid of larger tumors with normal PTT.^164^ Although it is widely accepted that combining methods enhances overall efficacy, the combination of PTT and chemotherapy has been the focus of extensive investigation. Several chemotherapy agents, particularly DOX, have been shown to increase the clinical benefit of PTT. This is despite research suggesting that chemo-PTT altered to activate anti-tumor immunity can have considerable therapeutic effects against primary and metastatic cancers.^165,166^ In a study done by Wang *et al.*, they designed cyclic arginyl-glycyl-aspartic acid (C-RGD) loaded with (DOX) doxorubicin GQDs, where C-RGD acted as a targeting agent (ligand), and DOX acted as a chemotherapy agent. Their results revealed that synergetic therapy inhibits tumor growth and is performed efficiently *in vitro* with good photothermal efficiency and obtained satisfactory pH-responsive drug-release behavior.^167^ Similarly, Fang *et al.* prepared hollow mesoporous carbon nanoparticles with GQDs combinational therapy with a regular size of 120 nm, which exhibited pH/NIR-controlled drug release behavior with negligible *in vivo* toxicity. Additionally, NIR irradiation has improved the antitumor suppression of nanoparticles.^168^ Furthermore, Kao and colleagues reported *in vivo* results demonstrating the efficacy of the as-produced GQDs in killing cancer cells while exhibiting a uniform shape, smaller distribution, and superb biocompatibility.^169^

### C. GQDs with PTT and immunotherapy

**FIG. 11.**
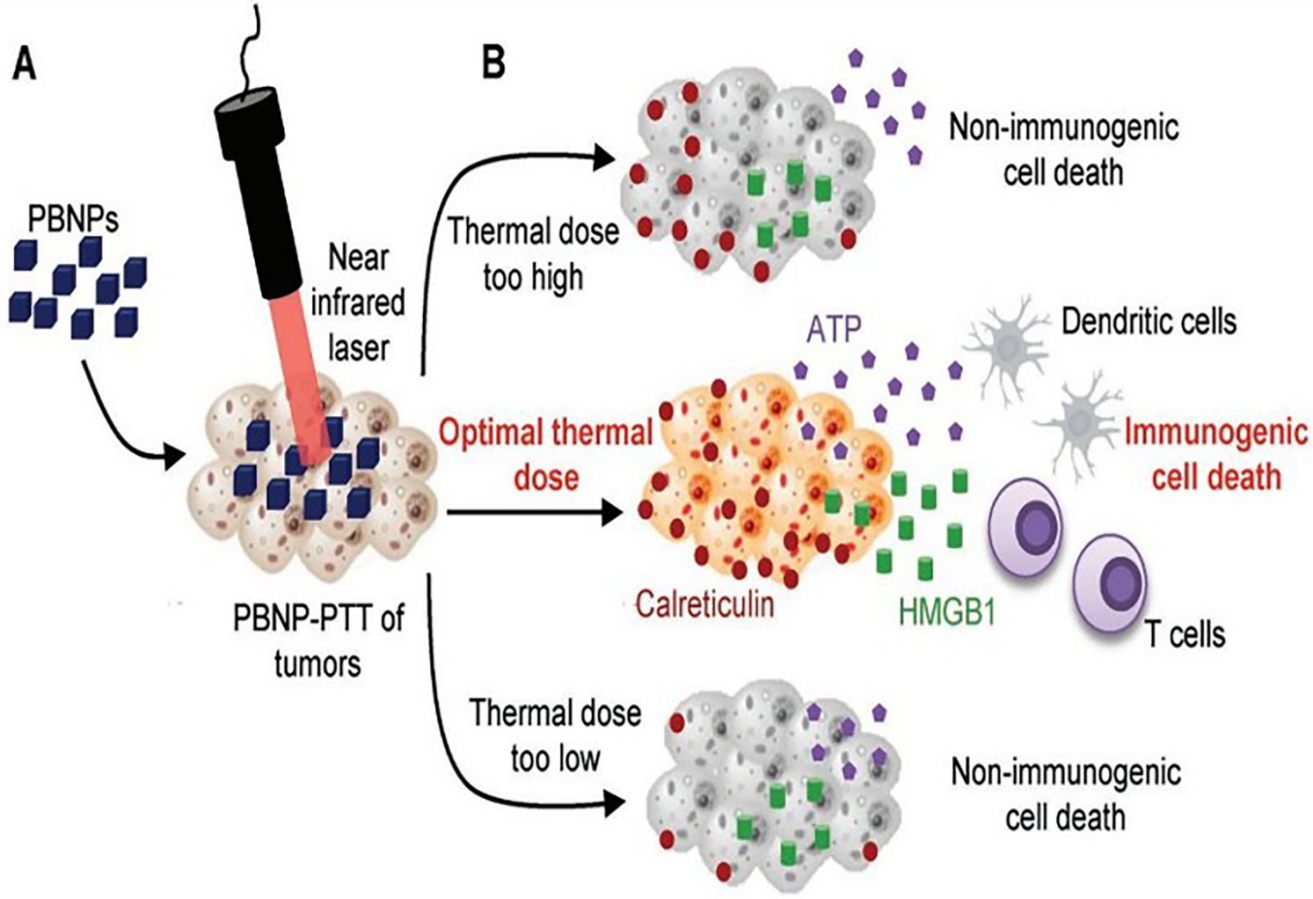
(a) Immunogenic cell death generated by PTT when optimal thermal dose administered with Prussian blue nanoparticle (PBNP). (b) When PBNP-PTT is delivered at the optimum temperature range (thermal dosage), tumor cells die immunogenically, releasing ATP, HMGB1, and surface calreticulin. These actions induce a strong antitumor immune response that improves therapeutic treatment. Reprinted with permission from Sweeney *et al.,* Small **14**, 1800678 (2018). Copyright 2018 John Wiley and Sons Clearance Center, Inc.^174^

Immunotherapy, which utilizes the immune system of the host to identify and eliminate cancer cells, is usually advised for cancer treatment. Recent studies focus on the immunological response following nanomaterial-based PTT. Figure 11 illustrates the mechanism of PTT-induced immunogenic cell death, wherein tumor cells can be destroyed by PTT after nanodrugs are directly or indirectly targeted to the tumor site and tumor-associated antigens and cell fragments are released, activating systemic immunity and eradicating residual or metastatic cancer.^170^ In addition, the development of immunological memory may help to avoid cancer reappearance. Photothermal immunotherapy is a type of PTT that uses nanomaterials to destroy tumors and promote long-term anticancer immune responses. Also, combining PTT with immunotherapy can improve the effectiveness of treatment for both primary tumors and cancer cells that have spread to other parts of the body.^171^ According to a study, PTT based on gold nanoshells was found to enhance the inflammatory cytokines, inorganic nanomaterials, and chemokines as well as the development of dendritic cells within the tumor-draining lymphatic system, giving rise to the priming of antitumor CD8+ effector T cell feedbacks. This treatment, combined with tumor-specific T cells that were transplanted, effectively inhibited tumor growth at distant sites and prevented lung metastases from spreading.^172^ Xia *et al.* explored the GQD layers efficiently protected partial miR155 from enzymatic degradation when the RNA was cross-linked with the GQDs via disulfide linkages, providing an experimental basis for the safe delivery of miRNAs *in vivo*.^173^

### D. GQDs with PTT and photodynamic therapy

PTT and photodynamic therapy (PDT) are both light-induced medicinal procedures. Due to oxidative stress, PDT drugs would be activated by light and produce singlet oxygen, which is lethal to adjacent cells. In PDT, the NIR area is widely appreciated in terms of penetration, comparable to PTT. PDT and PTT have recently received a lot of interest in treating a variety of illnesses including malignant tumors, due to their low severity.^175,176^ Therefore, combining PTT with PDT in a combinational therapy could be an effective cancer treatment. Some PTT agents, such as gold nanoparticles, could act as both PTT ablation agents and PDT carriers at the same time.^177^ In a study conducted by Wo *et al.*, multiple therapies were combined at once for cancer treatment, including mechanical, magnetic, photodynamic, photothermal, and chemotherapies, where GQDs decorated with hollow magnetic nanospheres, also coated with silica shells (HMNS/SiO_2_/GQDs), were again loaded with the anticancer drug doxorubicin (DOX), which proved that the multimodal system killed cancer cells with four different synergetic effects.^178^ Ashkbar *et al.* prepared high crystalline GQDs with withered leaves by hydrothermal synthesis and combined PDT and PTT and demonstrated excellent laser stability and monitoring structural changes. Furthermore, research on combinational therapy found that the nanocomposites + PDT + PTT technique could be a suitable alternative for chemotherapy in curing breast tumors.^179^ Furthermore, Liu and colleagues demonstrate an improved synergistic PTT and PDT effect on killing non-small-cell lung cancer (NSCLC) without toxicity, and the treatment removes tumor xenografts *in vivo* with no apparent negative outcomes.^180^

### E. GQDs with PTT and radiotherapy

**FIG. 12.**
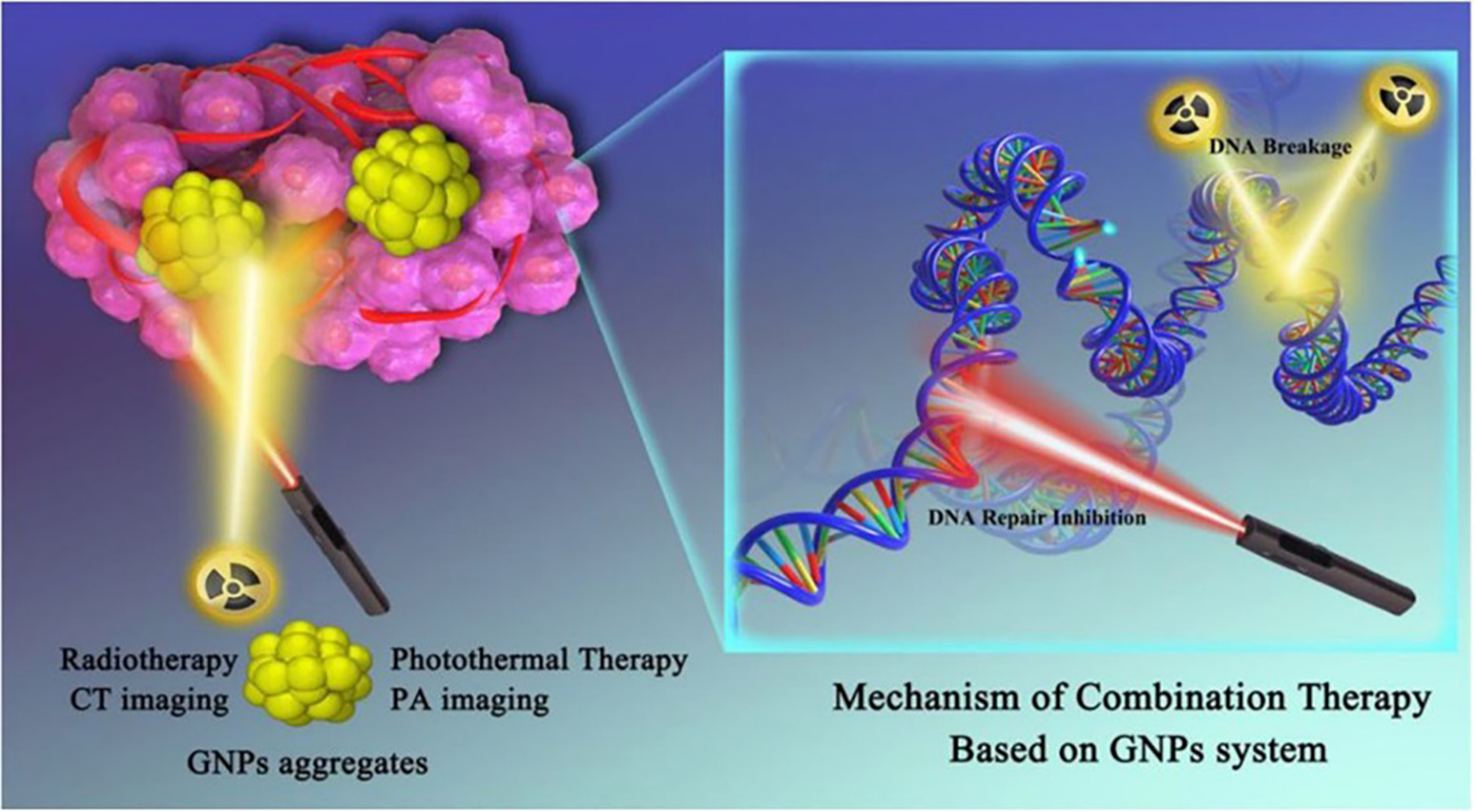
Mechanism of this combination therapy at the DNA level. Reprinted with permission from Zhang *et al.,* Nanomedicine **29**, 15499634 (2020). Copyright 2020 Elsevier Clearance Center, Inc.

Photo-radiotherapy is a type of combinatorial therapy that combines phototherapy and radiation therapy methods to improve clinical outcomes. The success of radiotherapy (RT) is dependent on the presence of oxygen molecules in the tumor area. However, in solid tumors, hypoxia and insufficient blood vessels limit RT efficacy. The soft tissues of tumors can absorb a small amount of radiation. As a result, under normal conditions and with safe radiation doses, RT efficacy is modest. However, a study using some metallic nanostructures as radiosensitizers demonstrated increased x-ray absorption and better RT efficacy.^181^ Zhang *et al.* demonstrated the use of a gold nanoparticle system in RT and PTT, illustrated in Fig. 12, in which DNA damage and DNA repair inhibition were inserted to damage DNA, resulting in optimal synergetic therapy, which was sited more precisely with the use of multimodal imaging. The radiation efficiency was increased, and the side effects were reduced.^182^ Ruan *et al.* synthesized GQDs with a variety of oxygen-containing groups to improve tumor radiation efficacy, and they observed different cell cycle alterations such as apoptosis, cellular arrest, and reduction of cell growth. They also looked into whether irradiation produces an excess supply of reactive oxygen species in tumor cells, resulting in mitochondrial dysfunction.^183^ In a preclinical model, Mishra *et al.* developed a nanomaterial prospective PTT application to control radiation- and chemo-resistant tumors. This study adds a value on the potential influence of PTT for *in vivo* ablation, the efficacy of which is closely related to PTT dose optimization.^184^ As a result, combining PTT and RT has a lot of potential for uncovering the unexplained causes of major illnesses.

## VIII. SUMMARY AND OUTLOOK

Scientists have made significant advances in GQDs with photothermal therapy and a tremendous revolution in the biomedical field over the last decade. GQDs have wonderful biological properties like functional groups, fluorescence, low toxicity, and biocompatibility, while photothermal therapy has other advantages such as being minimally invasive, target-specific, and controllable. As a result, the combination is appropriate for cancer therapy. The minimal cytotoxicity *in vitro* and *in vivo*, remarkable fluorescence in bioimaging, efficient photothermal conversion efficiency, and excellent cell viability of these two combinations have proved an adequate cancer therapy. Meanwhile, it is well understood that the small size of GQDs plays a pivotal role inside organs such as the kidney, spleen, and liver.

Similarly, earlier *in vivo* studies were not satisfactory due to the accumulation of nanoparticles inside the organs, despite that new results are promising. The optimal range of the NIR range is 808 nm, which is used in most of the studies, where the NIR-II window can be lucrative with different experiments. Moreover, surface functionalization and heteroatom doping of GQDs can open new ways for cancer therapy. The use of GQDs and photothermal therapy with other combinational therapy by decorating compatible elements have opened up vast possibilities, thereby having tremendous synergetic effects for the elimination of tumors. The presence of an oxygen functional group on GQDs helps in other combinational therapies like drug loading and gene therapies. Based on the rapid progress in the fields of nanomaterials, nanotechnology, and nanomedicine, we believe GQDs with photothermal therapy would be a brilliant probe for precision and accuracy, and they play a key role in theranostics. In conclusion, this review has provided the developments of GQDs and photothermal therapy, together with the latest fabrication methods, different combinational therapies used, and *in vitro* and *in vivo* mechanisms with a basic understanding of those contexts and also suggested the future direction for the development of GQDs and photothermal therapy.

## Data Availability

No data were used for the research described in the review article.
